# Effect of an acute session of intermittent exercise on trimethylamine N-oxide (TMAO) production following choline ingestion

**DOI:** 10.1007/s11306-024-02177-0

**Published:** 2024-10-05

**Authors:** Marilyn L.Y. Ong, Christopher G. Green, Samantha N. Rowland, Katie Rider, Harry Sutcliffe, Mark P. Funnell, Andrea Salzano, Liam M. Heaney

**Affiliations:** 1 School of Sport, Exercise and Health Sciences, Loughborough University, Loughborough, LE11 3TU UK; 2 Exercise and Sports Science Programme, School of Health Sciences, Universiti Sains Malaysia, Health Campus, Kubang Kerian, Kelantan, 16150 Malaysia; 3 Present Address: Diabetes Research Centre, NIHR ARC East Midlands, University of Leicester, Leicester, LE5 4PW UK; 4 Department of Translational Medical Sciences, “Federico II” University, Naples, Italy; 5 Cardiology Unit, AORN A Cardarelli, Naples, Italy

**Keywords:** Biomarker, Cardioprotection, Cardiovascular Risk, Exercise, Gut Microbiome, Nutrition

## Abstract

**Introduction:**

Trimethylamine N-oxide (TMAO) is a gut bacteria-dependent metabolite associated with poor cardiovascular health. Exercise is a known cardioprotective activity but the impact of an acute bout of exercise on TMAO production is unknown.

**Objectives/Methods:**

This study assessed choline-derived production of TMAO following a single bout of intermittent exercise in a young, healthy cohort.

**Results:**

Choline supplemented after either exercise or a time-matched resting period demonstrated a similar increase in circulating TMAO across an 8-hour period.

**Conclusion:**

This suggests that a single bout of intermittent exercise does not alter gut microbial metabolic behaviour and thus does not provide additional cardioprotective benefits related to blood levels of TMAO.

**Supplementary Information:**

The online version contains supplementary material available at 10.1007/s11306-024-02177-0.

## Introduction

The gut microbiota is known to be associated with cardiovascular disease (CVD) via the conversion of dietary compounds into downstream metabolites (Heaney, 2020). Trimethylamine N-oxide (TMAO) is produced via the metabolism of trimethylamine-containing compounds (e.g., choline and carnitine) and its presence in circulation has been heavily linked to the development and severity of several different CVD conditions (Thomas & Fernandez, 2021). There is, therefore, a growing interest in how lifestyle factors can influence the production/presence of TMAO and thus mediate/attenuate its negative associations with cardiovascular health (Coutinho-Wolino et al., 2021; Thomas & Fernandez, 2021). Physical activity and exercise represent the most important lifestyle intervention known to improve CVD (Tucker et al., 2022), with single bouts of exercise showing clinically relevant cardioprotection for around 2–4 h (Thijssen et al., 2022). As exercise causes alterations in the gut environment (e.g., a temporary redistribution of blood flow from the gastrointestinal tract) (Brouns & Beckers, 1993), it is of interest to understand the impact of exercise on the emergence of circulating TMAO levels. Indeed, any potential reductions in dietary-induced TMAO levels mediated by acute bouts of exercise could offer a positive benefit of including exercise routines prior to meal intake to add further to the known cardioprotective aspects of exercise.

Although preliminary reports on the effect of mid-to-long term exercise have been published (Erickson et al., 2019), no data exist indicating the effects of acute exercise on TMAO production. Therefore, the aim of the current study was to determine the effect of a single bout of exercise on serum TMAO concentrations in healthy adults following ingestion of a commercially available choline health supplement.

## Materials and methods

Eleven healthy and physically active participants volunteered and completed the study (six female, five male; age 24 ± 3 yrs (mean ± standard deviation); height 1.77 ± 0.08 m; body mass 74.5 ± 10.5 kg; body mass index 23.9 ± 2.5 kg/m^2^). All experimental testing was approved by the Loughborough University Human Ethics Review Sub-Committee (Ref: 4835). Participants were provided with details of the study in plain English and provided written informed consent. Participants performed two main trials (following a familiarisation visit) which included either an intermittent exercise protocol or a time-matched resting period (seated within the laboratory environment). The exercise consisted of six bouts of 3-min running at 70% heart rate reserve interspersed with 3-min walking, with 5-min warm-up and cool-down sessions. Full details of the trial protocols are available in the supplementary material. Participants were asked to refrain from exercise and high TMAO-precursor foods in the 24 h before each main trial.

Blood samples were collected via insertion of a cannula into an antecubital vein, drawn into serum tubes (S-Monovette, Sarstedt Ltd, Leicester, UK), and left on ice to clot for 30 min prior to centrifugation. Following the exercise protocol or rest period, participants ingested 700 mg of choline in the form of choline bitartrate (2 × 350 mg; Solgar, Leonia, NJ, USA) in hydroxypropyl methyl cellulose capsules as a compound known to raise TMAO levels (Cassambai et al., 2019). Venous blood samples were collected immediately prior to supplement ingestion (0 h) and at 2, 4, 6, and 8 h post-ingestion. Blood samples were immediately stored at -80 °C until analyses for serum TMAO (Heaney et al., 2016) and choline levels by liquid chromatography-tandem mass spectrometry (LC-MS/MS).

Statistical analyses were performed using STATA MP (v17, StataCorp, Texas, TX, USA) and IBM SPSS Statistics (v28, IBM Corp, Armonk, NY, USA) on log-transformed data. A linear mixed-effects model of concentrations on time by trial interaction with random intercepts by participant was used, applying a false discovery rate (FDR) of 5% on post-hoc contrasts using the Benjamini-Hochberg method. All comparisons which passed the threshold set by the 5% FDR are reported as the unadjusted *p* value. Full details of the analytical and statistical procedures are available in the supplementary material.

## Results and discussion

**Fig. 1:**
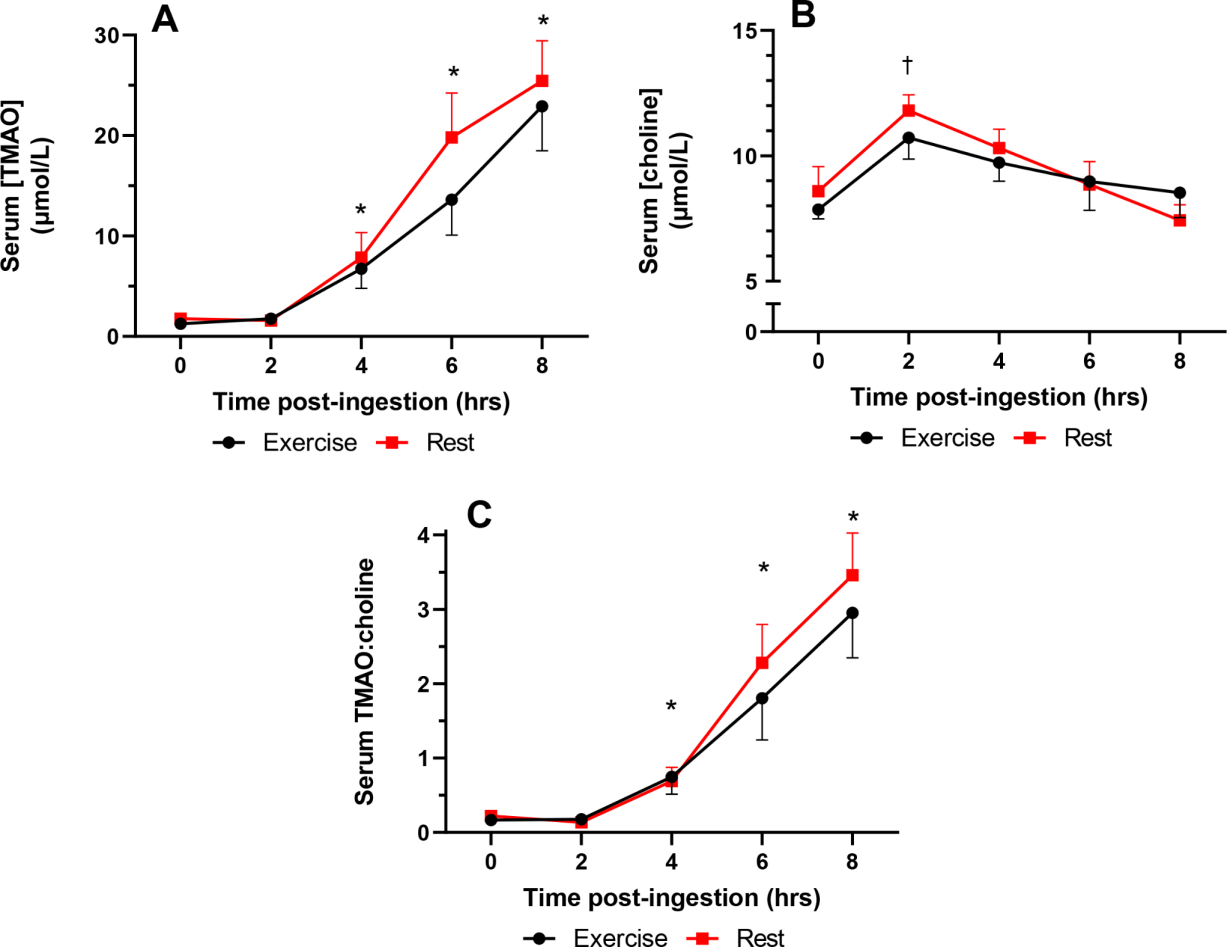
(**A**) Acute changes in serum TMAO levels over 8 h following choline supplementation. (**B**) Acute changes in serum choline levels over 8 h following choline supplementation. (**C**) Acute changes in serum TMAO: choline over 8 h following choline supplementation. All data are presented as means with error bars showing the standard error. * indicates greater than 0 h within each respective trial (*p* < 0.001). † indicates greater than 0 h within each respective trial (*p* ≤ 0.002)

In both exercise and rest trials, circulating TMAO concentrations were observed to rise across the 8-hr period following supplementation, with increased levels compared to 0 h at 2 h (both trials *p* < 0.001), 4 h (both trials *p* < 0.001), 6 h (both trials *p* < 0.001), and 8 h (both trials *p* < 0.001) (Fig. 1A). Despite a visual trend for a minor decrease in TMAO levels in the exercise trial, no statistical differences were identified between trials (*p* ≥ 0.212). Similarly, when the total area under the curve (tAUC) values were calculated and compared between trials, no differences were observed (*p* = 0.261). Furthermore, in both trials, circulating choline levels were observed to rise transiently and return to baseline across the 8-hr period following supplementation, with increased levels compared to 0 h noted only at 2 h post-supplementation for exercise and rest trials (*p* ≤ 0.002). No differences in serum choline levels compared to 0 h were noted from the 4 h timepoint onwards (*p* ≥ 0.055) (Fig. 1B). No differences were identified between trials (*p* ≥ 0.594). Similarly, when the tAUC were calculated and compared between trials, no differences were observed (*p* = 0.560). In order to assess the acute rise of TMAO levels in conjunction with the circulatory uptake of choline, TMAO: choline was calculated. Similarly to the TMAO data alone, in both exercise and rest trials, TMAO: choline values were observed to rise across the 8-hr period following supplementation, with increased levels compared to 0 h at 2 h (both trials *p* < 0.001), 4 h (both trials *p* < 0.001), 6 h (both trials *p* < 0.001), and 8 h (both trials *p* < 0.001) (Fig. 1C). Despite a visual trend for a minor decrease in TMAO levels in the exercise trial, no statistical differences were identified between trials (*p* ≥ 0.134).

To our knowledge, this is the first study to investigate the influence of an acute bout of intermittent exercise on choline-induced production of TMAO. The results demonstrated that a single bout of intermittent exercise in young, recreationally active, and otherwise healthy individuals did not alter the acute phase gut bacteria-mediated conversion of choline (i.e., within 8 h) when compared to a time-matched resting period. Mean concentrations of serum TMAO at 8 h increased to similar levels in the exercise and rest trials (mean values of 22.9 vs. 25.4 µmol/L, respectively), with the largest mean delta between trials seen at 6 h (13.6 vs. 19.8 µmol/L); although, the variation in response of TMAO production between participants meant that no overall difference could be confirmed. This is likely due to the small sample size and thus a lack of statistical power. Additionally, as TMAO levels were still observed to be rising in both cohorts at 8 h post-ingestion, a longer period of study (e.g. up to 12–16 h) may provide more insight into whether any deviation of production is observed/maintained. Notably, it has been suggested that TMAO levels decrease after 12 weeks of exercise (Erickson et al., 2019), suggesting that any effect of exercise on circulating TMAO is not acute (as per our protocol), but prolonged exercise training is required to observe a positive reduction in TMAO concentration. Importantly, the level of TMAO production which occurs following precursor ingestion is dependent on the gut microbial profile (Zhu et al., 2020), which may have led to an increased inter-individual response for TMAO in the current study. Further understanding of the gut microbial composition, via faecal sampling, could have provided evidence to understand the homogeneity/heterogeneity of our participant cohort in terms of microbiome content. Indeed, it has been shown that the intensity of exercise (i.e., combined aerobic and resistance moderate intensity continuous training, or combined aerobic and resistance high-intensity interval training) differentially affects bacterial relative abundance within the gut, along with aspects of microbial metabolic function (Torquati et al., 2023). This could infer that alternative acute exercise protocols (e.g. longer, more intense, or combined modalities) may result in different outcomes to our data. Nonetheless, the present study adds to the knowledge of the relationship between exercise and TMAO, being the first to investigate the effect of acute intermittent exercise on gut-mediated production of TMAO. Further studies are necessary to fully elucidate any potential role of exercise on gut microbial metabolism.

In conclusion, this study demonstrated that an acute, single bout of intermittent exercise did not alter gut bacterial behaviour with respect to the metabolism of a post-exercise dietary choline intake. These data suggest that a single bout of exercise does not provide immediate/transient protection from the potential cardiovascular risk of elevated levels of circulating TMAO, albeit demonstrated in a young, healthy cohort which may not be fully reflective of the situation in those at risk of CVD.

## Electronic supplementary material

Below is the link to the electronic supplementary material.

Supplementary Material 1

## Data Availability

Raw data relating to the TMAO and choline analyses can be found at 10.17028/rd.lboro.25202246.
