# Supplementary material for: Effect of an acute session of intermittent exercise on trimethylamine N-oxide (TMAO) production following choline ingestion
Source: Metabolomics. 2024 Oct 5;20(5):110. doi: 10.1007/s11306-024-02177-0 (PMC11455687; doi:10.1007/s11306-024-02177-0)
Supplement: Supplementary file 1 — Supplementary Material 1 [file 11306_2024_2177_MOESM1_ESM.pdf]

SUPPLEMENTARY MATERIAL

Effect of an acute session of intermittent exercise on trimethylamine N-oxide (TMAO) production following choline ingestion

Marilyn L.Y. Ong, Christopher G. Green, Samantha N. Rowland, Katie Rider, Harry Sutcliffe, Mark P. Funnell, Andrea Salzano, Liam M. Heaney

Exercise and Resting Trial Protocol

During the first visit to the laboratory, participants completed a health screen questionnaire, provided written informed consent, and were familiarised with all experimental procedures including the equipment, and exercise protocol being used for the main trials. Two main intervention trials (exercise or rest) were performed in a randomised repeated-measures crossover experimental design. Main trial day visits were separated by at least seven days. Participants were asked to maintain their habitual dietary intake throughout the study but to avoid red meat, seafood, eggs, and dairy in the 24 hrs prior to and during each study visit. All food and fluid consumed in the day prior to the first intervention visit was recorded and participants were asked to replicate this on the day prior to the following intervention visit. In addition, participants were asked to refrain from caffeinated beverage intake and strenuous exercise (light physical activity, e.g. walking, was permitted) in the 24 hrs prior to each study visit and were asked to arrive at the laboratory following an overnight fast (≥10 hrs). All blood samples were collected following 10 min of supine rest via a cannula inserted into an antecubital forearm vein. At the beginning of the trial, participants consumed a standardised breakfast consisting of two pieces of toasted white bread with strawberry jam (Tesco, UK) and 500 mL of plain water. One hour following breakfast, participants performed either the intermittent exercise protocol or a time-matched rest period.

The exercise protocol began with a 5-min warm-up at 25% of HR reserve (HRR) on a motorised treadmill (h/p/cosmos mercury® 4.0, Nussdorf-Traunstein, Germany), where HRR is age-predicted HRmax – resting HR (Karvonen et al. 1957; Tanaka et al. 2001). Participants then completed six sets of intermittent exercise involving three minutes of running at 70% of HRR, interspersed by three minutes of walking at 30% of HRR. This was followed by a 5-min cool down at 25% of HRR (total exercise time of 46 min). The speed of the treadmill was

automatically adjusted based on the HR (±10 beats/min) transmitted to the treadmill receiver from a chest worn HR monitor (H7, Polar Electro, Finland). The intensity of the exercise was calculated as follows: Warm-up: [25% x (age-predicted HRmax – resting HR)] + resting HR; Exercise: [70% x (age-predicted HRmax – resting HR)] + resting HR; Cool-down: [25% x (age-predicted HRmax – resting HR)] + resting HR. The exercise trial design was a modified approach to a previously published protocol (Taylor et al. 2017).

Following the exercise protocol or rest period, participants ingested 700 mg of choline in the form of choline bitartrate (2 x 350 mg; Solgar, Leonia, NJ, USA) in hydroxypropyl methyl cellulose capsules. Venous blood samples were collected immediately prior to supplement ingestion (0 hrs) and at 2, 4, 6, and 8 hrs post-ingestion. All samples were drawn into 4.5 mL serum tubes (S-Monovette, Sarstedt Ltd, Leicester, UK) containing a clot activator and remained on ice for 30 min prior to centrifugation (2500 ×g, 20 min, 4°C). Serum samples were stored at -80°C prior to analysis. Participants were free to complete light daily activities (e.g. walking) between the post-exercise sampling timepoints and were instructed to refrain from consuming high-choline foods for the remainder of the day. A list of high-choline content foods was provided to all participants to identify food groups/types to be avoided during this period.

Sample Analyses

All solvents (water, methanol, and acetonitrile) were of LC-MS grade and purchased from Fisher Scientific (Loughborough, UK) or VWR International (Lutterworth, UK). TMAO (98.9% purity) and choline chloride (≥99% purity) were purchased from Merck (Gillingham, UK), with D9-TMAO (>98% purity) and D9-choline chloride (>98% purity) from Cambridge Isotopes (Tewksbury, MA, USA). Formic acid and ammonium hydroxide were purchased from Fisher Scientific.

Serum TMAO and choline were quantified by liquid chromatography-tandem mass spectrometry (LC-MS/MS). All LC-MS/MS data were processed using MassLynx 4.1 software (Waters Corp., Milford, MA, USA). An Acquity UPLC liquid chromatograph was coupled to Quattro Ultima triple quadrupole mass spectrometer (Waters Corp.) operated in multiple reaction monitoring (MRM) mode. Briefly, stable isotope dilution was performed by mixing 20 μL of sample with 80 μL of 10 μmol/L D9-TMAO or 5 μmol/L D9-choline chloride in

methanol. The sample was then vigorously mixed on a vortex and the protein fraction pelleted by centrifugation (21,100×g, 10 min, 4°C) and the resulting supernatant transferred to a low-volume sample vial.

For TMAO, analyses were performed according to a previously validated method (Heaney et al. 2016) using a UPLC BEH HILIC column (130 Å, 1.7 µm, 2.1 mm x 100 mm; Waters Corp.) and pre-column (Acquity VanGuard; 130 Å, 1.7 µm, 2.1 mm x 5 mm; Waters Corp.) heated to 50 °C with a solvent flow rate maintained at 600 µL/min. The sample injection volume was 5 µL. Solvent A was 0.025% ammonium hydroxide and 0.045% formic acid (pH 8.1), and solvent B was pure acetonitrile. The gradient started at 95% B and reduced linearly to 4% B in 0.8 min before returning to 95% B at 1.9 min until 2.5 min. MRM was performed by electrospray ionisation in positive ionisation mode using precursor ions of *m/z* 76.1 (TMAO) and *m/z* 85.1 (D9-TMAO) with collision energies at 20V and 25V for product ions of *m/z* 58.1 and *m/z* 66.1, respectively. Samples were quantitated based on the ratio of peak areas for TMAO to D9-TMAO and compared to a calibration curve. Based on duplicate sample measurements, the mean precision for TMAO in study samples was 1.4%.

Analyses to assess free circulating choline were performed using a UPLC BEH HILIC column (130 Å, 1.7 µm, 2.1 mm x 100 mm; Waters Corp.) and pre-column (Acquity VanGuard; 130 Å, 1.7 µm, 2.1 mm x 5 mm; Waters Corp.) heated to 50 °C with a solvent flow rate maintained at 600 µL/min. The sample injection volume was 5 µL. Solvent A and B were as described for the TMAO analysis. The gradient started at 80% B and reduced linearly to 4% B in 0.8 min before returning to 80% B over 1.1 min and held for 0.6 min. MRM was performed by electrospray ionisation in positive ionisation mode using precursor ions of *m/z* 104.1 (choline) and *m/z* 113.2 (D9-choline) with collision energies at 17V for product ions of *m/z* 60.2 and *m/z* 69.2, respectively. MS source conditions were as follows: capillary voltage 3 kV, cone voltage 35V, source temperature 120°C, desolvation temperature 500°C, cone gas flow 250 L/hr, and desolvation gas flow 900 L/hr. Samples were quantitated based on the ratio of peak areas for choline to D9-choline and compared to a calibration curve. Assay validation experiments demonstrated the assay to have a mean accuracy of 102% and precision of 2.4% across a 0.1 to 100 µmol/L range (Table S1). Based on duplicate measurements, the mean precision for choline in study samples was 0.8%.

Table S1. Validation data including precision and accuracy for serum choline assessed by LC-MS/MS.

Concentrations (µmol/L)	Precision (RSD)	Accuracy
0.1	9.0%	127%
0.5	2.4%	118%
2	0.2%	96%
5	1.1%	93%
10	1.5%	92%
20	1.5%	94%
50	1.3%	90%
100	1.8%	103%
**Mean**	**2.4%**	**102%**

Note: Precision was calculated as the relative standard deviation (RSD) across all injections at each calibration point. Accuracy was determined as the back-calculated concentration from the mean value of each calibration point.

Statistical Analyses

Statistical analyses were performed using STATA MP (v17, StataCorp, Texas, TX, USA) and IBM SPSS Statistics (v28, IBM Corp, Armonk, NY, USA). All data were investigated for normality through the Shapiro-Wilk test and data were analysed in their log-transformed state. Analyses to understand the acute dynamics of serum TMAO and choline levels post-supplementation (i.e. from 0 to 8 hrs) were performed using a linear mixed-effects model of concentrations on time by trial interaction with random intercepts by participant. The model was computed using the restricted maximum-likelihood (REML) estimation with small sample inference on time by trial interaction using the Kenward-Rogers degrees of freedom method. Where a positive effect was identified, post-hoc contrasts of model estimates were computed and a false discovery rate (FDR) of 5% was applied using the Benjamini-Hochberg method. All comparisons which passed the threshold set by the 5% FDR are reported as the unadjusted *p* value. Total area under the curve (tAUC) data for acute levels of TMAO and choline were calculated using cubic splines via the *pkcollapse* command in STATA MP. Paired sample t-tests were used to compare tAUC across trials. An alpha value (*p*) threshold of 0.05 was applied.

References

Heaney, L. M., Jones, D. J. L., Mbasu, R. J., Ng, L. L., & Suzuki, T. (2016). High mass accuracy assay for trimethylamine N-oxide using stable-isotope dilution with liquid chromatography coupled to orthogonal acceleration time of flight mass spectrometry with multiple reaction monitoring. *Analytical and Bioanalytical Chemistry*, *408*(3), 797–804. https://doi.org/10.1007/s00216-015-9164-6

Karvonen, M. J., Kentala, E., & Mustala, O. (1957). The effects of training on heart rate; a longitudinal study. *Annales medicinae experimentalis et biologiae Fenniae*, *35*(3), 307–15.

Tanaka, H., Monahan, K. D., & Seals, D. R. (2001). Age-predicted maximal heart rate revisited. *Journal of the American College of Cardiology*, *37*(1), 153–156. https://doi.org/10.1016/S0735-1097(00)01054-8

Taylor, J., Keating, S. E., Leveritt, M. D., Holland, D. J., Gomersall, S. R., & Coombes, J. S. (2017). Study protocol for the FITR Heart Study: Feasibility, safety, adherence, and efficacy of high intensity interval training in a hospital-initiated rehabilitation program for coronary heart disease. *Contemporary Clinical Trials Communications*, *8*, 181–191. https://doi.org/10.1016/j.conctc.2017.10.002
